# Lactic Acid Bacteria With Antioxidant Activities Alleviating Oxidized Oil Induced Hepatic Injury in Mice

**DOI:** 10.3389/fmicb.2018.02684

**Published:** 2018-11-06

**Authors:** Xiangna Lin, Yongjun Xia, Guangqiang Wang, Yijin Yang, Zhiqiang Xiong, Fang Lv, Wei Zhou, Lianzhong Ai

**Affiliations:** ^1^Shanghai Engineering Research Center of Food Microbiology, School of Medical Instrument and Food Engineering, University of Shanghai for Science and Technology, Shanghai, China; ^2^Yangzhou Yangda Kangyuan Dairy Ltd., Yangzhou, China

**Keywords:** Lactic acid bacteria, antioxidant, oxidized oil, oxidative damage, hepatic injury, nuclear factor erythroid 2-related factor 2

## Abstract

In order to screening new Lactic acid bacteria (LAB) strains to alleviating liver injury induced by oxidized oil, we isolated and screened LAB from Chinese fermented foods. *Lactobacillus plantarum* AR113, *Pediococcus pentosaceus* AR243, and *Lactobacillus plantarum* AR501 showed higher scavenging activity of α, α-Diphenyl-β-Picrylhydrazyl (DPPH) free radical and hydrogen radical, stronger inhibition of lipid peroxidation, and better protective effect on yeast cells *in vitro*. *In vivo*, oral administration of *L. plantarum* AR501 improved the antioxidant status of injury mice induced by oxidized oil including decreasing lipid peroxidation, recovering activities of antioxidant enzymes. Meanwhile, the gene expression of Nuclear factor erythroid 2-related factor 2 (Nrf2) of *L. plantarum* AR501 group was markedly elevated, and several antioxidant genes such as glutathione S-transferase (GSTO1), heme oxygenase-1 (HO-1), Glutamate cysteine ligase (GCL), and NAD(P)H:quinone oxidoreductase-l (NQO1) were subsequently up regulated in mice liver. Therefore, *L. plantarum* AR501 could be considered as potential candidates for production of functional foods that can alleviate the oxidative damage induced by oxidized oil.

## Introduction

In industrialized countries, heated or fried foods play an important role in diet (Koch et al., 2007). Vegetable oils are more popular due to their cholesterol-lowering effect, such as peanut oil, soybean oil and olive oil. However, compared to animal fats which are predominantly saturated except for fish oil and hence do not react with oxygen, vegetable oils are more reactive in an aerobic environment (Naz et al., 2004). The polyunsaturated fatty acids (PUFA) are rich in vegetable oil and they are labile compounds to form lipid peroxidation under the promoting effect of air, heat, light, and moisture (Eshak et al., 2010). As we all known, ingestion of oxidized oil is lead to a variety of diseases such as atherosis and cancer (Ibrahim et al., 1997; Eder, 1999; Hwang et al., 2000; Totani et al., 2008). It is reported that feeding oxidized oil to chicken, which results in induced oxidative stress in live birds and increased lipid and protein oxidation in breast muscle (Zhang et al., 2011).

Lactic acid bacterial (LAB) were reported have many benefits such as antimicrobial activity, ability to modulate immune response, improving in gastrointestinal (GI) ecosystems (Kullisaar et al., 2002; Cleusix et al., 2007; Persichetti et al., 2014; Tang et al., 2016). They are normalinhabitants of gastrointestinal tract of humans and animals. Several evidence also confirmed that *Lactobacillus casei*, *Lactobacillus acidophilus*, *Lactobacillus plantarum*, and *Lactobacillus delbreukii* have been used as model probiotics. They are used as dietary supplements for their benefits such as immunomodulatory nature and regulating gastrointestinal functions (Amdekar et al., 2013). Increasing attentions had been paid to the probiotic characteristics of LAB such as their antioxidant and anti-colorectal cancer (Lee et al., 2005; Kumar et al., 2012; Gao et al., 2013). However, LAB alleviating oxidized oil induced oxidative stress injury had never been reported.

In our previous study, LAB were screened by the tolerance of hydrogen peroxide after isolated from fermented cabbage and Chinese rice wine of different area. In this study, antioxidative properties of LAB were investigated by determining the DPPH and hydroxyl radicals scavenging effect and lipid peroxidation inhibiting effect. Moreover, we assessed protective effects of selected LAB against hydrogen peroxide toxicity on yeast. Subsequently, we evaluated effects of selected LAB on ameliorating oxidized oil induced liver damage *in vivo*. Enzyme activities of superoxide dismutase (SOD), glutathion peroxidase (GSH-Px), catalase (CAT), total antioxidant capacity (T-AOC) and the content MDA in serum and liver were detected. Meanwhile alanine transaminase (ALT), aspartate transaminase (AST) in serum were also determined. Subsequently, the relationship between ameliorating oxidative stress and Nuclear factor erythroid 2-related factor 2 (Nrf2) signaling pathway was determined.

## Materials and Methods

### Bacterial Strains and Culture Conditions

*L. plantarum* AR113, *P. pentosaceus* AR243, *L. plantarum* AR269, *L. plantarum* AR300, and *L. plantarum* AR501 were used in this study. They were previously screened by tolerance of hydrogen peroxide up to 1.5 mM (Lin et al., 2018). All strains were isolated on de Man, Rogosa, and Sharpe (MRS) agar plates and stored in MRS broth with 20% glycerol at −80°C. For all subsequent experiments, the strains were propagated in MRS broth at 37°C for 16 h anaerobically and serially transferred at least twice prior to use in this study. The intact cells were obtained by centrifugation (10,000 *g* for 5 min at 4°C), and then washed by sterilized isotonic saline (0.9%) for three times. The final concentration of intact cells was adjusted to 1.0 × 10^9^ CFU/mL.

### Assay Antioxidant Activity of LAB Strain *in vitro*

#### Scavenging of α, α-Diphenyl-β-Picrylhydrazyl (DPPH) Free Radical

The scavenging effects on DPPH of LAB were measured by a modification of method utilized by Kao and Chen (2006). 2.0 mL of intact cells were added to 1.0 mL of ethanolic DPPH radical solution (0.02 mM). The mixtures were incubated at room temperature in the dark for 30 min after mixed vigorously. The controls used deionized water instead of intact cells, and the blanks used anhydrous ethanol instead of DPPH. The absorbance of supernate were measured in triplicate at 517 nm after centrifugation at 6,000 *g* for 10 min. The scavenging ability was defined as follows:

$$\begin{array}{l} \mathrm{Scavengingrate}(\%)=[1-(\mathrm{Asample}-\mathrm{Ablank})/\mathrm{Acontrol}]\\ \;\times 100\% \end{array}$$

#### Scavenging of Hydroxyl Radical

The hydroxyl radical scavenging assay were conducted by a Fenton reaction method (Li et al., 2012). Briefly, the reaction mixture containing 1.0 mL of FeSO_4_ (5 mM), 1.0 mL of ethanolic salicylic acid (5 mM), 1.0 mL of H_2_O_2_ (3 mM), 1.0 mL of intact cells and 6 mL of distilled water were incubated at 37°C in water bath for 15 min after mixing. The controls used distilled water instead of intact cells and the absorbance of resulting solution were measured in triplicate at 510 nm after centrifugation at 6,000 *g* for 10 min. The absorbance changes of reaction mixture indicated the scavenging rate of LAB for hydroxyl radical. Hydroxyl radical scavenging rate is expressed as follows:

Scavengingrate(%)=[(1−Asample/Acontrol]×100%

#### Lipid Peroxidation Inhibition Activity of LAB in a Liposome System

Inhibition of LAB on linolenic acid peroxidation was measured by the 2-thiobarbituric acid (TBA) method according to Lin and Chang (2000) with small modifications. The linolenic acid emulsion was kept in the dark at 4 °C for 1 h after mixed with 1 mL linoleic acid, 0.2 mL Tween 80 and 18.8 mL distilled water. To assay the antioxidant activity of LAB, 0.5 mL PBS (0.2 M, pH 7.4), 1 mL linoleic acid emulsion, 0.2 mL FeSO_4_ (0.01%), 0.2 mL H_2_O_2_ (0.56 mM) and 0.5 mL intact cells were mixed and incubated at 37 °C water bath for 12 h. Distilled water was used in the blank samples. The reaction mixture were incubated at 100 °C for 30 min after mixed with 0.2 mL trichloroacetic acid (TCA, 4%), 2 mL thiobarbituric acid (TBA, 0.8%), and 0.2 mL butylated hydroxytoluene (BHT, 0.4%) and then allowed to cool. The absorbance were measured at 532 nm after centrifugation at 1,000 *g* for 10 min. The percentage of inhibition capacity of LAB on the linoleic acid peroxidation was calculated as follows:

$$\begin{array}{l} \mathrm{Inhibitionrateoflipidperoxidation}(\%)=[1-\mathrm{Asample}/\mathrm{Ablank}]\\ \;\times 100\% \end{array}$$

#### Yeast Live Cell Model

The protective effect of LAB against oxidative toxicity in eukaryotic cells was measured by the method of Kuda et al. (2014) with some modifications. *Saccharomyces cerevisiae* BR30 (CGMCC10378) belonged to our lab was incubated with Malt Extract Broth (MEB) at 28°C for 14 h with shaking at 180 rpm. The LAB were centrifuged at 6,000 *g* for 10 min after incubated with MRS broth at 37°C for 18 h. The LAB intact cells were prepared in previous method. The intact cells suspension were then sterilized at 100°C water bath for 20 min. *S. cerevisiae* BR30 culture (0.5 mL) was inoculated into 5 mL of sterilized LAB suspension in triplicate. After 1 h of incubation at 28°C with shaking at 180 rpm, H_2_O_2_ (final concentration 30 mM) was added to the suspension. After 1 h of incubation at 28°C with shaking, serial dilutions were immediately made and the surviving yeast cells were counted with Malt Extract Ager (MEA) at 28°C for 48 h. Survival rate was calculated as the ratio of CFU of surviving cells to the CFU without hydrogen peroxide.

### Protect Effects of LAB on Oxidized Oil Treated Mice

#### Animals and Animal Treatments

All animal procedures for this study were performed in compliance with guidelines of the national “Laboratory Animals Regulations,” “Shanghai Regulations of Laboratory Animal Management” and Institutional Animal Care and Use Committee at Shanghai Jiao Tong University (Shanghai, China). The experimental protocols were approved by the Institutional Animal Care and Use Committee at Shanghai Jiao Tong University (No. 201801130). Male ICR mice with an average body weight of 20 ± 1 g (purchased from Shanghai SLRC Laboratory Animal Co., Ltd.) were randomly assigned to six treatment groups of 10 mice each. Normal group was fed with standard diet with 10% normal oil, and model group was fed with standard diet with 10% oxidized oil. Positive group was fed with standard diet with 10% oxidized oil +6% taurine. Experimental groups (different LAB): mice were fed with standard diet with 10% oxidized oil + different LAB. Mice in the positive and experimental groups were treated with taurine and LAB (1.0 × 10^9^ CFU/mL) at a dose of 10 mL kg^−1^ once daily, respectively. The mice were housed in an air-conditional room maintained at 23 ± 2°C at a relative humidity of 50 ± 10%, and a hours light/dark cycle. The standard diet was composed of 18% crude protein, 5% fiber, 4% fat, 50% carbohydrate, 10% water, vitamins and minerals. The soybean oil was obtained from supermarket and heated at 160°C for 3 days to oxidization. The oxidized oil was characterized as follows: peroxide value (POV) 20.5 mM/kg oil, acid value (AV) 6.1 mg/g oil. Body weight was determined every week. After feeding for 10 weeks, mice were sacrificed.

#### Measurement of Organ Index

The Heart, liver, brain, spleen, kidneys, lungs, thymus, and pancreas were weighted after dissected and washed with ice-cold normal saline immediately. Organ index were calculated by their weights relative to the final body weight.

#### Preparation of Serum and Liver Homogenate

After the final administration for 24 h, blood sample were collected from the orbital sinus. Serum sample were obtained via centrifugation 1,150 *g* for 10 min at 4°C and stored at −80°C for further analysis. The liver tissues of each animals were homogenized with pre-cold normal saline solution. The homogenates were centrifuged at 1,150 *g* for 10 min at 4°C and stored at −80°C for further analysis.

#### Biochemical Assays

The activities of ALT, AST, CAT, GSH-Px, SOD, and MDA level were measured according to the commercial kit (Nanjing Jiancheng Bioengineering Institute, Nanjing, China). The protein concentration was determined using a total protein assay kit (Nanjing Jiancheng Bioengineering Institute, Nanjing, China) using bovine serum albumin as standard. All procedure were performed in duplicate with three parallel samples.

#### Gene Expression

The total RNA of liver tissue in each group were extracted using TRIzol reagent (Sangon Biotech) according to the manufacturer’s instructions and then reverse transcribed into cDNA using RT reagent Kit with gDNA Eraser (Takara, China) according to the manufacturer’s instructions. The cDNA were used as the template for further quantitative RT-PCR analysis. A lightcycler real-time PCR system and the SYBR Green PCR Kit were used to conduct real-time PCR. Each sample had been measured in triplicate. The conditions of conducting the PCR reaction included: at the initial stage, denaturing at 95°C for 30 s, followed by 40 cycles of denaturing at 95°C for 5 s, annealing and extension at 60°C for 30 s. The primers of Nrf2, NAD(P)H:quinone oxidoreductase-l (NQO1), heme oxygenase-1(HO-1), glutamate–cysteine ligase catalytic subunit (GCLc), glutamate-cysteine ligase regulatory subunit (GCLm), and glutathione S-transferase (GSTO1) were designed by using Primer 6 software (PREMIER Biosoft company, Palo Alto, CA, United States) (Supplementary Table S1), and β-actin was employed as a house-keeping gene. To allow comparisons of mRNA expression levels, the real-time PCR data were analyzed by using the 2^−ΔΔCT^ assay and expressed as fold change relative to expression in the normal group.

### Statistical Analysis

Tests were carried out in triplicate for all experiments. All data were presented as mean ± standard deviations (SD). Statistical comparison were made using the statistical software package SPSS 22.0. Significant differences between treatments were tested by analysis of variance (ANOVA) followed by a comparison between treatments performed using Duncan and Fisher’s least significance difference (LSD) method, with levels of significance of *p* < 0.05.

## Results

### Antioxidant Activity of LAB *in vitro*

*In vitro*, antioxidant activities of intact cells at 1.0 × 10^9^ CFU/mL of the 5 strains were showed in Table 1. The scavenging effects of LAB on DPPH radicals were range from 5.73 to 35.72%, and *L. plantarum* AR113 exhibited the highest scavenging capability. The scavenging effect on hydrogen radicals is one of the important index of antioxidation. There was a wide variability to remove hydrogen radicals of LAB in Table 1. *L. plantarum* AR113 exhibited the highest scavenging capability in all strains with scavenging rate of 49.60%; *L. plantarum* AR501 had degrading activity, while *L. plantarum* AR269 exhibited the lowest removal activity of 7.08%. Furthermore, both strains were exhibiting inhibition of lipid peroxidation to different degrees. The inhibition rates of lipid peroxidation ranged from 23.95 to 46.94%, the strongest inhibition rate was 46.94% for *L. plantarum* AR113, *L. plantarum* AR269, *L. plantarum* AR501 had comparable degrading capability and *L. plantarum* AR300 showed the lowest inhibition rate for 23.95%.

**Table 1:** Antioxidant activity of LAB *in vitro.*

	*L. plantarum* AR113	*P. pentosaceus* AR243	*L. plantarum* AR269	*L. plantarum* AR300	*L. plantarum* AR501
DPPH scavenging rate (%)	35.72 ± 1.24^a^	10.32 ± 1.19^c^	11.81 ± 0.53^c^	5.73 ± 0.47^d^	21.03 ± 1.41^b^
Hydrogen radicals scavenging rate (%)	49.60 ± 0.34^a^	26.45 ± 1.38^c^	7.08 ± 0.16^e^	14.63 ± 1.25^d^	31.33 ± 1.70^b^
Inhibition rate of lipid peroxidation (%)	46.94 ± 8.90^a^	35.25 ± 3.57^b^	39.09 ± 2.10^ab^	23.95 ± 8.96^c^	41.08 ± 8.12^ab^
Survival rate (%)	79.91 ± 4.63^a^	38.34 ± 2.74^c^	51.30 ± 1.83^b^	38.03 ± 3.67^c^	52.42 ± 3.42^b^

*Means with different letters in the same row differ significantly (p < 0.05).*

Though *S. cerevisiae* cells have a certain tolerance to some stress such as oxidative stress (Sousa-Lopes et al., 2004) and ethanol. However, 30 mM hydrogen peroxide decreased the survival of *S. cerevisiae* BR30 cells to 21.86% during 1 h. *S. cerevisiae* BR30 cells was distinctly protected by sterilized LAB intact cells. Sterilized *L. plantarum* AR113 showed the strongest protective effect against H_2_O_2_ on *S. cerevisiae* BR30 for 79.91%, *L. plantarum* AR269 *and L. plantarum* AR501 presented a preferable protective effect on *S. cerevisiae* BR30, while *L. plantarum* AR300 and *P. pentosaceus* AR243 also protected *S. cerevisiae* BR30 (Table 1). Among the five LAB strains, *L. plantarum* AR113, AR501 and *P. pentosaceus* AR243 with better antioxidant properties *in vitro* were further subject to *in* *vivo* studies.

### Protective Effect of LAB on Oxidized Oil Treated Mice

#### Changes of General Appearance, Body Weight, and Organ Index

During the whole experiment period, no death occurred and there were significantly difference in body weight among all groups (*p* > 0.05) (data not shown.)

The liver index of model group increased significantly as compared with normal mice (*p* < 0.05), while Taurine and *L. plantarum* AR501 treated mice decreased significantly (*p* < 0.05) compared to model group (Table 2). The brain index of *P. pentosaceus* AR243 group increased significantly (*p* < 0.05) from model group, however other groups were no significantly difference compared to model group. The spleen and kidney index of *L. plantarum* AR113 group decreased significantly (*p* < 0.05) as compared with model group. The thymus index of the *L. plantarum* AR113 group increased significantly (*p* < 0.05) compared to model group. However, there was no significant different between other group and model group (*p* > 0.05).

**Table 2:** Effect of LAB on the organ index.

	Thymus	Spleen	Kidney	Pancreas	Liver	Brain
Normal group	0.15 ± 0.00	0.23 ± 0.03	1.14 ± 0.21	0.64 ± 0.10	3.11 0.22^∗^	1.08 ± 0.06
Model group	0.13 ± 0.00	0.24 ± 0.02	1.35 ± 0.16	0.62 ± 0.08	3.36 0.07	1.04 ± 0.06
Positive group	0.15 ± 0.04	0.23 ± 0.04	1.25 ± 0.16	0.83 ± 0.08^∗^	3.17 0.14^∗^	1.13 ± 0.03
AR113 group	0.17 ± 0.02^∗^	0.18 ± 0.05^∗^	1.09 ± 0.18^∗^	0.77 ± 0.05	3.19 0.03	1.10 ± 0.11
AR243 group	0.13 ± 0.00	0.20 ± 0.05	1.23 ± 0.11	0.72 ± 0.06	3.35 0.09	1.15 ± 0.05^∗^
AR501 group	0.15 ± 0.02	0.20 ± 0.01	1.19 ± 0.18	0.65 ± 0.03	3.17 0.21^∗^	1.11 ± 0.18

*Each value is expressed as mean ± standard, ^∗^ means significantly different compared to model group (p < 0.5).*

#### Effect of LAB on Lipid Peroxidation Level

**FIGURE 1:**
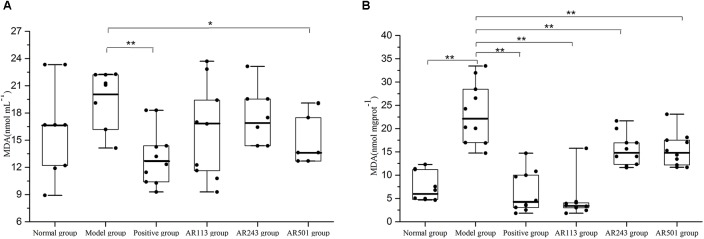
Effect of LAB on oxidized oil induced levels of lipid peroxidation in serum **(A)** and liver **(B)** of mice. Value are expressed as means ± SD (*n* = 10), ^∗^*p* < 0.05 vs. model group. ^∗∗^*p* < 0.01 vs. model group.

MDA as a typical byproducts of lipid peroxidation has been shown to be an efficient biomarker for oxidative stress (Nielsen et al., 1997). Levels of lipid peroxidation in serum and liver of model group were significantly higher than normal group, which indicates the model is successful (Figure 1). In Figure 1A, the ability of *L. plantarum* AR501 to reduce level of lipid peroxidation in serum was significantly compared to model group (*p* < 0.05). We found that levels of lipid peroxidation of LAB groups (*L. plantarum* AR113, AR501 and *P. pentosaceu*s AR243) in liver were significantly decreased (*p* < 0.01) compared to model group (Figure 1B). Moreover, *L. plantarum* AR113 reduced level of lipid peroxidation as good as positive group, and make it return to normal level in liver (Figure 1B).

#### Effect of LAB on Serum Aminotransferase (ALT and AST) Levels

**FIGURE 2:**
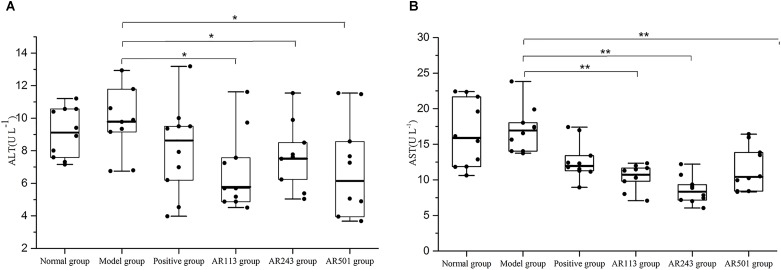
Effect of LAB on serum enzyme activities of **(A)** alanine aminotransferase (ALT) and **(B)** aspartate aminotransferase (AST) levels. Where U/L means the biological value of ALT or AST in per liter serum. Values are expressed as mean ± SD (*n* = 10), ^∗^*p* < 0.05 vs. model group, ^∗∗^*p* < 0.01 vs. model group.

ALT and AST are known as indicator enzymes of liver injury. Injury to liver cells often results in a leak of ALT and AST into the bloodstream and then elevates the serum aminotransferase levels. ALT and AST levels in serum of different groups after feeding as described above were shown in Figure 2. Higher level of AST than ALT were founded in the present study, which in accordance with primer findings (Tian et al., 2015). A small increase of ALT and AST levels was observed in model group compared to normal group. It was showed that ALT levels were decreased significantly after feeding LAB compared to model group (*p* < 0.05) in Figure 2A. Similar result was obtained from Figure 2B. Furthermore, AST levels dropped to lower than positive group in AR113 and AR243 groups. Thus, LAB provide a protective effect on oxidized oil induced liver injury.

#### Effect of LAB on Antioxidative Status in Serum

**FIGURE 3:**
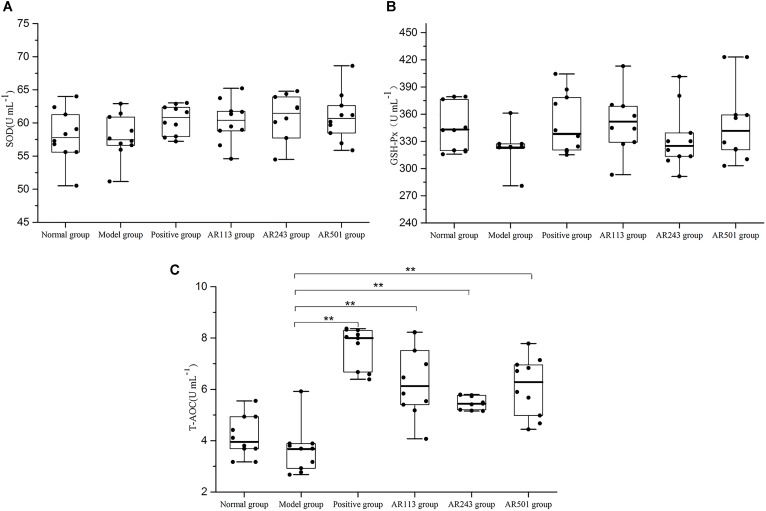
Effect of LAB on oxidized oil induced changes in the activity of SOD **(A)**, GSH-Px **(B)** and T-AOC **(C)** in serum of mice. Where U/L means the biological value of SOD, GSH-Px or T-AOC in per milliliter serum. Values are expressed as mean ± SD (*n* = 10), ^∗^*p* < 0.05 vs. model group, ^∗∗^*p* < 0.01 vs. model group.

It is accepted that GSH-Px, CAT, and SOD are three of the most important physiological antioxidants against free radical, and they can prevent lipid peroxidation, subsequently (Nielsen et al., 1997; Wang et al., 2008; Wang and Yang, 2010). In this study, we detected several key antioxidant enzymes including SOD, GSH-Px, and T-AOC. Model group exhibited significantly decreased the activities of T-AOCon which the LAB groups showed effective protection (Figure 3C), which suggesting that administration of LAB may inhibit oxidized oil induced oxidative stress by elevating antioxidants. However, no significant difference of activities of SOD (Figure 3A) and GSH-Px (Figure 3B) was observed among these groups.

#### Effect of LAB on Antioxidative Status in Liver

**FIGURE 4:**
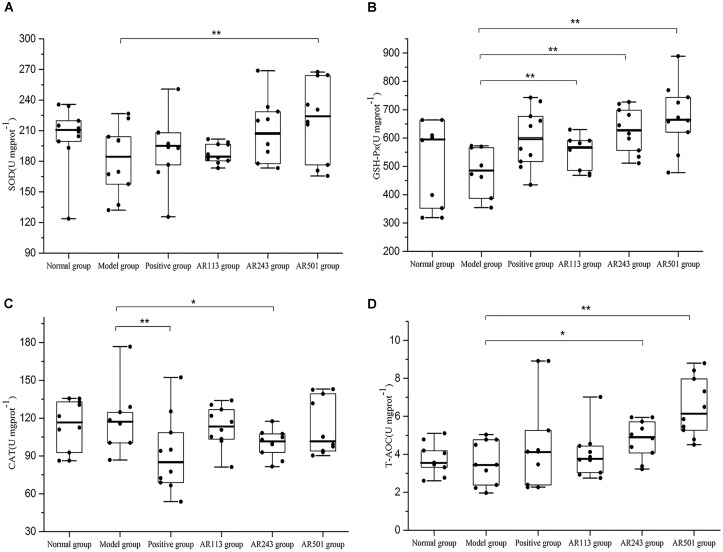
Effect of LAB on oxidized oil induced changes in the activity of SOD **(A)**, GSH-Px **(B)**, CAT **(C)**, and T-AOC **(D)** in liver of mice. Where U/mgprot means the biological value of SOD, GSH-PX, CAT or T-AOC in per milligram protein in liver. Values are expressed as mean ± SD (*n* = 10), ^∗^*p* < 0.05 vs. model group, ^∗∗^*p* < 0.01 vs. model group.

Next, we chose liver as the representative organ to detect the oxidative stress related parameters. Oxidized oil treatment affected the activities of SOD, GSH-Px, CAT, and T-AOC level in liver. The results showed that *L. plantarum* AR113, AR501, *and P. pentosaceu*s AR243 alleviated oxidized oil induced liver injury in different degrees in Figure 4. Feeding with *L. plantarum* AR501, the activities of SOD and GSH-Px and T-AOC levels were significantly increased (*p* < 0.01) compared to model group, confirming the antioxidative protection of the *L. plantarum* AR501. Feeding with *P. pentosaceu*s AR243 significantly increased the activities of GSH-Px and T-AOC (*p* < 0.05) compared to feeding with normal saline. However, there were only SOD activity increased significantly in AR113 group compared to model group.

#### Effect of LAB on Gene Expression in Liver

**FIGURE 5:**
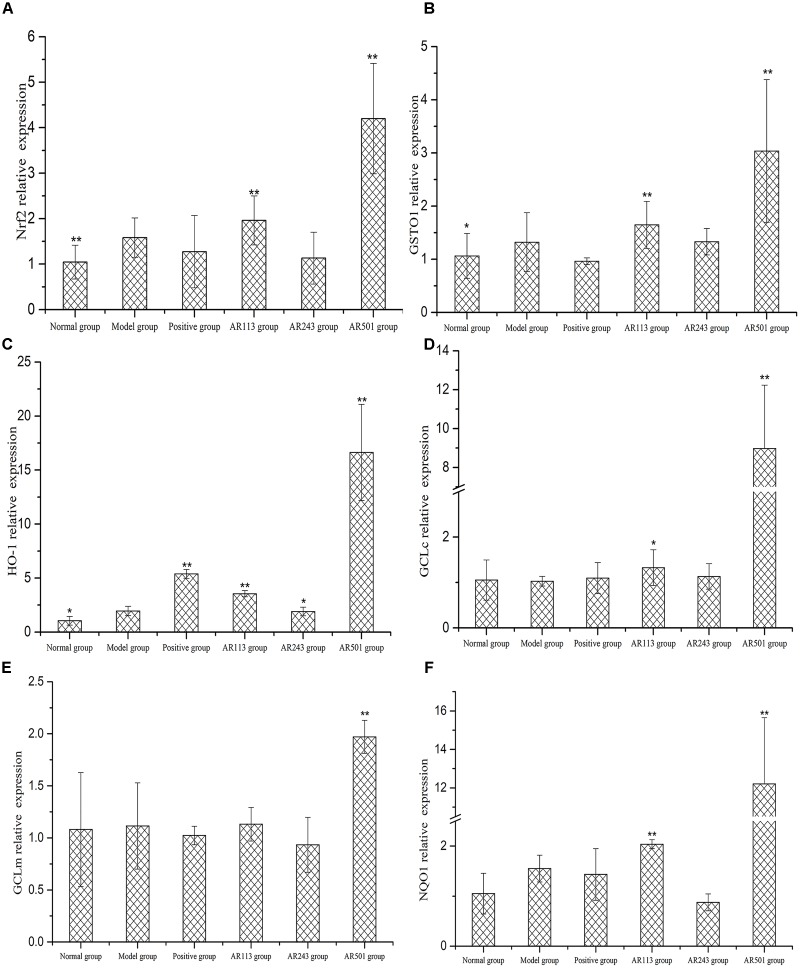
Effect of LAB on oxidized oil induced changes of gene expression of Nrf2 **(A)**, GSTO1 **(B)**, HO-1 **(C)**, GCLc **(D)**, GCLm **(E),** and NQO1 **(F)** in liver of mice. Values are expressed as mean ± SD (*n* = 10), ^∗^*p* < 0.05 vs. model group, ^∗∗^*p* < 0.01 vs. model group.

In order to further study the mechanism of LAB in the regulation of these antioxidant enzymes, we assessed the expressions of related antioxidant gene. The gene expression of Nrf2, GSTO1, HO-1, GCLc, GCLm, and NQO1 were quantified using real time PCR with β-actin as reference gene. The nuclear factor erythroid 2-related factor 2 (Nrf2; encoded by Nfe2l2 gene) is a transcription factor responsible for the regulation of cellular redox balance and protective antioxidant and phase II detoxification responses in mammals. Our results showed that the expression of Nrf2 were markedly increased in AR113 and AR501 groups as compared to model group (*p* < 0.01), whereas the expression in normal group was decreased significantly compared to model group (Figure 5A). Upon activation, Nrf2 is translocated to the nucleus where it binds to ARE and induces the expression of a number of antioxidative and phase II detoxification enzymes including GSTO1, HO-1, GCLc, GCLm, and NQO1. The expressions of GSTO1, HO-1, GCLc, GCLm, and NQO1 mRNA were significantly increased after feeding *L. plantarum* AR501 as compared to these of model group (*p* < 0.01) (Figures 5B,C). In *L. plantarum* AR113 group, the expression of GSTO1, HO-1, GCLc, and NQO1 mRNA were significantly increased as compared to that of model group (Figures 5B,C). These findings suggested a general correlation of the protective effects of *L. plantarum* AR113 and AR501 with Nrf2 and the activation of related antioxidant enzymes (Figure 5).

## Discussion

In human body, many physiological and biochemical processes may produce reactive oxygen species (ROS) and produce oxygen centered free radicals as byproducts (Thibessard et al., 2004). Superabundant accumulate of ROSsuch as hydroxyl radicals, hydrogen peroxide, superoxide anion radicals, severely damage proteins, DNA and lipids, which led to further tissue damage and organ dysfunction (Halliwell and Gutteridge, 1984). Compared with hydroxyl radicals, hydrogen peroxide is a weak oxidant, but it may give rise to hydroxyl radical which cause oxidative damages of cells. Many *lactobacillus* strains were found to resist hydrogen peroxide to different extent (Kullisaar et al., 2002; Wu et al., 2009). In previous study, *L. plantarum* AR113, AR269, AR300, AR501, and *P. pentosaceus* AR243 exhibited strong resistance to hydrogen peroxide. DPPH has been used widely as a radical to evaluate the capacity of antioxidant (Antolovich et al., 2002). However, DPPH is stable in methanol solutions and is not a naturally occurring ROS. Hydroxyl radicals has been reported to be the most harmful ROS. It is formed by the Fenton action in the presence of metal ions such as ferrous and copper. Lipid peroxidation is one of the most commonly discussed oxidation reactions. Antioxidant enzymes are regarded as important enzymatic defense systems against oxidative stress in LAB. In addition, antioxidant activity of some LAB could ascribe to their production, such as exopolysaccharides (Pan and Mei, 2010), lipoteichoic acid (Yi et al., 2009) and cell-surface proteins (Li et al., 2012). In this study, *L. plantarum* AR113, AR501 *and P. pentosaceus* AR243 shown strong DPPH and hydroxyl radical scavenging and better inhibition of lipid peroxidation (Table 1). *S. cervisiae* BR30 are utilized as an individual organism for the study of replicative life span and chronological life span. Using yeast can be considered a simple biologicals model for the study of antioxidant differs from chemicals assays. Sterilized *L. plantarum* AR113 showed the strongest protective effect against H_2_O_2_ on *S. cerevisiae* BR30 for 79.91% (Table 2).

It is known that long-term intake of oxidized oil is harmful for our health such as induced liver injury and reduced diet palatability (Chao et al., 2001). Oxidized oil could lead to several alterations of lipid metabolism, which might be of physiologic relevance and it also decreases activities of antioxidant enzymes. Our study demonstrated that after fed with LAB, the body weight of mice was no different (data not shown) whereas, the organ index was changed in different degrees (Table 2).

MDA serves as a reliable maker of oxidative stress-mediated lipid peroxidation. Thus MDA level was used as a maker of oxidative stress-induced liver injury. The liver is a major organ involved in lipid metabolism. It was reported that *Lactobacillus plantarum* MA2, *Lactococcus lactis subsp. lactis* 12, and *Lactobacillus plantarum* C88 inhibited increase of MDA level in serum or liver induced by D-gal (Pan and Mei, 2010; Zhang et al., 2013; Tang et al., 2016). Our findings agreed with previous reports. Oral administration of LAB for 10 weeks significantly decreased the level of lipid peroxidation in the serum and liver of oxidized oil induced oxidative stress mice (Figure 1).

ALT and AST are known as indicator enzymes of the presence of liver disease. In this study, ALT and AST levels in serum was affected by oxidized oil which also meaning that oxidized oil induced liver injury. Oral administration of LAB significantly reduced the ALT and AST levels in serum of mice, indicating that the liver injury by oxidized oil could be ameliorated by LAB (Figure 2).

Normally, ROS can be controlled by endogenous free radical scavengers such as SOD, CAT, and the glutathione peroxidase/glutathione/glutathione reductase system. The defense system of antioxidant enzymes may reduce oxidative stress and alleviate oxidative-related disease (Yu et al., 2015). In present study, the activities of antioxidant enzymes in the mice serum and liver were significantly decreased by the treatment of oxidized oil. However, *L. plantarum* AR501 could markedly increase the activities of SOD in liver (Figure 4A). Moreover, *P. pentosaceus* AR243 and *L. plantarum* AR501 could significantly increase the activities of GSH-Px in liver (Figure 4B). These findings indicated that supplementation of LAB could enhance the activities of antioxidant enzymes in mice.

The total antioxidant capacity (T-AOC) reflects the capacity of the non-enzymatic antioxidant defense system (Zhang et al., 2013). In this study, obvious increase were observed in T-AOC level in both serum and liver after supplemented with *L. plantarum* AR501 (Figures 3C, 4D). Similar to our findings, Zhang et al reported that oral administration of *L. casei* Zhang significantly increased T-AOC in liver form hyperlipidemic rats (Zhang W. et al., 2011; Zhang Y. et al., 2010).

Nrf2 is the key molecule, which mediates the response of the endogenous antioxidant system (Gao et al., 2016). Recent studies indicate that activating the Nrf2/ARE pathway has a hepato-protective effect (Long et al., 2016). Under basal conditions, Nrf2 is bond to Keap1 in the cytoplasm, and Nrf2 dissociates from Keap1, transfers to the nucleus, binds to antioxidant response elements (ARE) and transactivates phase II detoxifying and antioxidant genes after cells expose to inducers such as oxidative stress (Ren et al., 2011). As enhancer elements, ARE initiate the transcription of a series of genes encoding by phase II enzymes such as GSTO1, HO-1, GCLc, GCLm, and NQO1. In this study, mice fed with oxidized oil only showed oxidative stress and liver injury. After treated with *L. plantarum* AR501, the gene expression of Nrf2 were increased. As for the regulation of Nrf2, the antioxidative enzymes such as GSTO1, HO-1, GCLc, GCLm, and NQO1 was promoted significantly, subsequently (Figure 5). As expected, *L. plantarum* AR501 caused the activation of Nrf2 in liver and then increased the genes of phase II enzymes expression, thus alleviating oxidized oil induced hepatic injury in mice.

## Conclusion

In order to screening new LAB strains with antioxidant property from traditional Chinese fermented foods, we isolate LAB *in vitro*. *In vitro*, *L. plantarum* AR113, AR501, and *P. pentosaceus* AR243 showed higher DPPH free radical and hydrogen radical scavenging activity, stronger inhibition of lipid peroxidation, and better protective effect on yeast cells. *In vivo*, the results indicated that oral administration of *L. plantarum* AR501 improved the antioxidants status of oxidized oil induced oxidative stress, including decrease lipid peroxidation, renewing the activities of SOD, GSH-Px, and enhance T-AOC level. Meanwhile, *L. plantarum* AR501 caused the activation of Nrf2 in liver and then increased the genes of phase II enzymes expression, thus alleviating oxidized oil induced hepatic injury in mice. Therefore, *L. plantarum* AR501 could be considered as a potential strains for further utilization in protection of oxidized oil induced liver injury.

## Ethics Statement

All animal procedures for this study were performed according to institutional and governmental regulations about the use of experimental animals.

## Author Contributions

XL interpreted the results; collected test data drafted the manuscript. FL and WZ assisted to complete the experiment. YX and LA designed the study. GW, ZX, and YY polished the manuscript.

## Conflict of Interest Statement

The authors declare that the research was conducted in the absence of any commercial or financial relationships that could be construed as a potential conflict of interest.
