# Supplementary material for: Lactic Acid Bacteria With Antioxidant Activities Alleviating Oxidized Oil Induced Hepatic Injury in Mice
Source: Front Microbiol. 2018 Nov 6;9:2684. doi: 10.3389/fmicb.2018.02684 (PMC6232458; doi:10.3389/fmicb.2018.02684)
Supplement: Supplementary file 1 [file Table_1.doc]

Table S1 Primers for real-time PCR analyses

| Gene | Primer Sequences | Product Size/bp |
| --- | --- | --- |
| Mus-Nrf2  (NM-010902.3) | F: 5’-TTGGCAGAGACATTCCCATTTG-3’  R: 5’-AAACTTGCTCCATGTCCTGCTCTA-3’ | 172 bp |
| Mus-GSTO1  (NM-010362.2) | F: 5’- TCAGCGACTGGAAGCATTGG -3’  R: 5’- TTTAAGTACTCGCGGTAGGTCTTGG -3’ | 138 bp |
| Mus-HO-1  (NM-010442.2) | F: 5’- TGCAGGTGATGCTGACAGAGG -3’  R: 5’- GGGATGAGCTAGTGCTGATCTGG -3’ | 144 bp |
| Mus-GCLc  (NM-010295.2) | F: 5’-CAGATATTGGATGGAGAGTAGA-3’  R: 5’-CAGCGGAATGAGGAAGTC-3’ | 137 bp |
| Mus-GCLm  (NM-008129.3) | F: 5’- AGTTGGAGCAGCTGTATCAGTGG -3’  R: 5’- TTTAGCAAAGGCAGTCAAATCTGG -3’ | 104 bp |
| Mus-NQO1  (NM-008706.5) | F: 5’-GACGCCTGAGCCCAGATATT-3’  R: 5’-AGGACCGTTGTCGTACATGG-3’ | 176 bp |
| Mus-β-actin  (NM-007393.5) | F: 5’-AATCGTGCGTGACATCAA -3’  R: 5’-GCTCGTTGCCAATAGTGA-3’ | 140 bp |
